# Supplementary material for: Mapping the evolution of fertility support policies in China: A content and instrumental analysis
Source: PLoS One. 2025 Oct 9;20(10):e0332137. doi: 10.1371/journal.pone.0332137 (PMC12510515; doi:10.1371/journal.pone.0332137)
Supplement: S1 Appendix — (ZIP) [file pone.0332137.s001.zip › S1 Appendix. 226 original policy documents/9-国家人口计生委关于加强泛长三角地区流动人口计划生育服务和管理区域联动协作的指导意见(FBM-CLI-4-132698).doc]

国家人口计生委关于加强泛长三角地区流动人口计划生育服务和管理区域联动协作的指导意见

**发布部门：** [国家人口和计划生育委员会(含国家计划生育委员会)(已撤销)](https://www.pkulaw.com/law/chl?Aggs.IssueDepartment=60304) [机构沿革](javascript:void(0))

**发文字号：**国人口发〔2010〕36号

**发布日期：**2010.05.24

**实施日期：**2010.05.24

**时效性：** [现行有效](https://www.pkulaw.com/law/chl?Aggs.TimelinessDic=01)

**效力级别：** [部门工作文件](https://www.pkulaw.com/law/chl?Aggs.EffectivenessDic=XE0303)

**法规类别：** [计划生育管理](https://www.pkulaw.com/law/chl?Aggs.Category=09705)

**专题分类：** [长三角一体化发展](https://www.pkulaw.com/law/chl?Aggs.SpecialType=024)

国家人口计生委关于加强泛长三角地区流动人口计划生育服务和管理区域联动协作的指导意见
（国人口发〔2010〕36号）

上海市、江苏省、浙江省、安徽省、江西省、河南省、湖北省人口计生委：
　　为认真贯彻落实《中共中央国务院[关于全面加强人口和计划生育工作统筹解决人口问题的决定](https://www.pkulaw.com/chl/5e7b01010a4102ffbdfb.html)》（中发〔2006〕22号）和国务院《[流动人口计划生育工作条例](https://www.pkulaw.com/chl/f6b2c35d55b7a40ebdfb.html)》，探索建立泛长三角地区“统筹管理、服务均等、信息共享、区域协作、双向考核”工作机制，加快形成区域“一盘棋”工作格局，现就加强泛长三角地区流动人口计划生育服务管理联动协作，提出如下意见：
　　**一、**加强泛长三角地区流动人口计划生育服务管理区域联动协作的重要意义
　　泛长三角地区包括上海、江苏、浙江、安徽、江西、河南、湖北七省（市），是我国第一大经济圈和支撑我国经济发展、提升国家竞争力的主要动力区域。泛长三角区域内的人口流动性强、流动量大，区域内各省（市）间工作的互补性要求更高，因此加强省际协作，强化区域内部整合，规范各级人口计生部门流动人口服务管理工作标准，搭建起包括信息交流、均等化服务、政策研究等方面的区域协作框架，使泛长三角区域率先形成“信息互通、服务互补、管理互动、责任共担”的局面，对于最终实现全国“一盘棋”将起到引领示范作用。

　　**二、**指导思想和基本原则
　　（一）指导思想。
　　以党的十七大精神为指引，坚持以科学发展观为统领，转变政府职能，坚持以人为本，推动泛长三角地区人口和计划生育服务管理联动发展，努力使泛长三角地区成为全国流动人口计划生育服务管理区域联动合作的先行区、示范区。
　　（二）基本原则。
　　1．以人为本，便民利民。
　　依据人口和计划生育法律法规和政策规定，结合政府职能转变，按照以人为本、便民利民原则，重点解决泛长三角各省（市）间流动人口计划生育服务管理方面的协调问题。
　　2．先易后难，分步实施。
　　按照泛长三角各省（市）《人口与计划生育条例》规定，依法履行部门职责，按照先易后难、分步实施的原则，各地联动协作，加快解决制约计划生育服务管理“一盘棋”的重点问题。
　　3．探索创新，联动发展。
　　探索创新人口和计划生育服务管理模式，运用信息化手段及时共享人口和计划生育服务管理信息，提高管理效率，降低管理成本，为泛长三角地区人口和计划生育服务管理“一盘棋”奠定基础。
　　4．互惠互利，多方共赢。
　　加强泛长三角地区人口计生部门间的工作联动，促进转变工作作风，通过各方密切协作，提高工作效率，真正使群众得到便利和实惠，形成互惠互利、多方共赢的局面。

　　**三、**强化为民意识，简化和规范行政事务办理程序
　　（一）流动人口生育手续办理。
　　流动人口夫妻拟在现居住地生育第一个子女、需要办理一孩生育手续的，现居住地人口计生部门要依据《[流动人口计划生育工作条例](https://www.pkulaw.com/chl/f6b2c35d55b7a40ebdfb.html)》，为其办理相关手续，户籍地要配合做好婚育情况证明办理工作。户籍地和现居住地要做好一孩生育手续办理的信息通报与反馈工作。
　　逐步建立两地协作的再生育审批制度。区域内跨省流动人口夫妻要求再生育的，户籍地人口计生部门应当按照当地规定为其办理再生育审批手续。两地婚姻夫妻（指“区域内跨省婚姻，婚后居住在一方户籍所在地的夫妻”，下同）要求再生育的，按照方便群众的原则，可以由任何一方户籍地的人口计生部门按照当地规定办理审批手续。
　　（二）《独生子女父母光荣证》发放。
　　区域内跨省流动人口夫妻，自愿终生只生一个子女的，双方户籍地在同一省市的，由户籍地省市人口计生部门向其颁发《独生子女父母光荣证》，并落实相关的计划生育奖励待遇。
　　两地婚姻夫妻自愿终生只生一个子女的，分别由各自户籍地人口计生部门发放《独生子女父母光荣证》，并由各自户籍地落实相关的计划生育奖励待遇。
　　（三）《流动人口婚育证明》办理。
　　户籍地人口计生部门应当在成年育龄妇女外出前，为其免费办理全国统一的《流动人口婚育证明》。户籍地和现居住地人口计生部门应当相互配合，为流动人口办理《流动人口婚育证明》提供便利。对于未在户籍地办理《流动人口婚育证明》的流动育龄妇女，户籍地人口计生部门应当允许当事人委托他人代为办理《流动人口婚育证明》。

　　**四、**强化协作，规范服务和管理
　　（一）强化区域流动人口公共服务。
　　全面落实流动人口免费享受国家规定的基本项目的计划生育技术服务。现居住地为流动人口提供与户籍人口同等的计划生育、优生优育、生殖健康、避孕药具等技术服务。做好流入已婚育龄妇女孕检服务工作，及时通过网络信息系统将流入已婚育龄妇女孕检等信息通报给户籍地。逐步实现计划生育基本公共服务均等化和免费基本项目计划生育技术服务全覆盖。
　　户籍地人口计生部门应当依法落实法律法规规定的各项奖励优惠政策，帮助解决留守家庭的实际困难和问题，保障流动人口计划生育合法权益。要积极探索分类管理、诚信管理、委托管理等模式，户籍地在接到现居住地人口计生部门通报避孕节育信息后，不得要求已婚育龄妇女返回户籍地接受避孕节育情况检查，也不得到现居住地开展避孕节育检查或者向流动人口收取任何费用。
　　户籍地应当遵守国家人口计生委《关于撤销各地跨省设立的流动人口计划生育管理站的通知》（计生厅〔2000〕121号）规定，不得到现居住地设立跨省工作站（管理站、服务站或孕检站等）。如为了加强对流向相对集中的本地流出人口的服务管理，可以按照现居住地为主，户籍地配合的原则，在征得现居住地同意后，委派相关人员在现居住地统一领导和管理下，协助现居住地对户籍地流入人口开展宣传、教育、信息通报及联络协调等工作。
　　（二）加强重点对象的计划生育管理工作。
　　现居住地人口计生部门应当建立流入育龄妇女登记制度，及时掌握其怀孕、生育、避孕节育等信息。发现怀孕的，应尽快通报户籍地人口计生部门，共同查清其是否符合生育政策规定，并加强有关服务，寓管理于服务之中。对于不符合规定怀孕的，户籍地和现居住地要协同配合，做好人口计生法律法规和政策的宣传教育，明确告知违法生育的法律后果，动员当事人采取补救措施，并落实免费计划生育技术服务及有关待遇。各级人口计生部门要积极创造条件，开展联合执法、集中服务等活动，努力控制和减少违法生育现象，稳定低生育水平。
　　（三）规范流动人口社会抚养费征收。
　　对于违法生育的流动人口，户籍地与现居住地人口计生部门应当互相配合，共同做好社会抚养费征收工作。社会抚养费征收，按照户籍地征收、现居住地配合的原则执行。两地婚姻夫妻违法生育的，由夫妻婚后经常居住一方的户籍地人口计生部门负责征收，另一方户籍地配合做好征收工作。经常居住地不能确定的，由女方户籍地人口计生部门负责征收。
　　户籍地人口计生部门决定对违法生育嫌疑人立案调查的同时，应向另一地人口计生部门发出《协助做好社会抚养费征收工作的函》，另一地人口计生部门应当积极配合做好调查取证等工作。
　　（四）加大联合打击“两非”行为的力度。
　　建立区域协作、齐抓共管、联合执法的工作机制，联合打击非医学需要的胎儿性别鉴定和选择性别的人工终止妊娠行为（以下简称“两非”）。对已发生的“两非”案件，由案件发生地的人口计生部门牵头联合公安、卫生、工商、食品药品监管等部门立案侦查，相关地区的人口计生部门配合查处。
　　（五）建立健全出生登记实名制。
　　各级人口计生部门要加强与卫生、公安等部门的协调配合，加大住院分娩和出生实名登记宣传力度，积极引导和鼓励孕妇到医疗保健机构住院分娩。建立健全出生登记实名制度，督促提供分娩服务的医疗保健机构按规定查验孕产妇及其配偶的身份证明，做好信息登记，定期通报出生人口信息，建立完善部门共享的人口信息管理系统。

　　**五、**强化措施，实现区域“一盘棋”
　　（一）建立联动协作长效机制。
　　建立泛长三角地区流动人口服务管理联动合作联席会议制度。联席会议由各省（市）人口计生委主任、分管流动人口工作的副主任和流动人口服务管理处以及相关职能处室负责人组成。每年召开一次联席会议，由各省（市）人口计生委主任轮流担任召集人。各省（市）人口计生委流动人口服务管理处指定1名工作人员担任联席会议联络员，负责日常工作接洽。
　　（二）建立流动人口服务管理网络化协作机制。
　　各地应在加快建设流动人口服务管理信息系统的基础上，依托国家PADIS 流动人口子系统，建立泛长三角流动人口计划生育服务管理信息化协作平台，开展流动人口信息实时查询、实时协查，实现婚育、避孕节育等信息的网络通报。做到跨省流动人口信息区域共享、在线交换、动态更新，为流动人口计划生育服务管理区域协作提供保障。
　　（三）建立区域协作年度通报制度。
　　各地要加强工作信息交流。轮值省每年年初在各省（市）提出的区域协作工作设想的基础上，制定泛长三角区域协作年度方案，各省（市）人口计生委按照方案内容，认真组织实施，稳步推进。年底，各省（市）人口计生委应当总结本省（市）流动人口服务管理和区域协作进展情况，深入分析存在的问题，提出进一步推进区域协作的工作对策建议，由轮值省人口计生委汇总后，形成泛长三角区域协作年度报告，上报国家人口计生委并通报区域内省（市）人口计生委。
　　各地人口计生部门要切实树立流动人口计划生育工作区域“一盘棋”思想，结合本地区实际，认真贯彻执行本意见的要求，通过日常工作沟通、联席会议等方式，及时解决区域协作中出现的问题，不断完善协作制度。

国家人口计生委办公厅
二○一○年五月二十四日

© 北大法宝：（[www.pkulaw.com](https://www.pkulaw.com/)) 专业提供法律信息、法学知识和法律软件领域各类解决方案。北大法宝为您提供丰富的参考资料，正式引用法规条文时请与标准文本核对。
欢迎查看所有[产品和服务](https://www.pkulaw.com/helps/2.html)。法宝快讯：[如何快速找到您需要的检索结果？](https://www.pkulaw.com/helps/70.html)[法宝](https://www.pkulaw.com/helps/69.html)V6有何新特色？

扫描二维码阅读原文

**原文链接：**<https://www.pkulaw.com/chl/2f302f2d9bbfb8a5bdfb.html>
